# Feasibility of implementing a culturally adapted Prolonged Grief Disorder scale in the mental healthcare system in Nepal

**DOI:** 10.1017/gmh.2021.29

**Published:** 2021-09-15

**Authors:** Yoona Kim, Asmita Ghimire, Molly E. Lasater, Brandon A. Kohrt, Pamela J. Surkan, Nagendra P. Luitel

**Affiliations:** 1 Department of International Health, Johns Hopkins Bloomberg School of Public Health, Baltimore, MD, USA; 2 BP Koirala Institute of Health Sciences, Dharan, Kathmandu, Nepal; 3 Department of Mental Health, Johns Hopkins Bloomberg School of Public Health, Baltimore, MD, USA; 4 Department of Psychiatry, George Washington University, Washington, DC, USA; 5 Transcultural Psychosocial Organization Nepal (TPO Nepal), Kathmandu, Nepal

**Keywords:** Implementation, measurement, Nepal, prolonged grief disorder, widows

## Abstract

**Background:**

Nepali widows have a high prevalence of mental disorders, including prolonged grief disorder (PGD). Despite the considerable needs that Nepali widows have for mental health services, resources for mental health in Nepal are limited, amplifying the importance of accurate screening and diagnosis. The objective of this study was to explore the feasibility of implementing a culturally adapted Prolonged Grief Scale (PG-12/17-N) and provide actionable recommendations for its implementation.

**Methods:**

Twenty-five mental health service providers in Kathmandu and Chitwan, Nepal were interviewed using a semi-structured guide based on selected constructs from the Consolidated Framework for Implementation Research. Qualitative data were inductively and deductively coded and analyzed to identify prominent themes.

**Results:**

Providers reported that the main advantages of the scale were the need to identify widows at risk, cultural relevance, easy language, and inclusion of detailed and specific symptoms. Perceived weaknesses included the complexity in response options and scoring, length, item redundancy, overlap with depression symptoms, and lack of somatic symptoms. Providers discussed the need for training, supervision, and a referral and detection system required to implement the scale in Nepal. Further development of a brief version of the scale as a routine screener may facilitate detection and referral to care.

**Conclusion:**

Based on the results showing need to address PGD in Nepali widows, further efforts are needed to increase awareness about PGD and develop evidence-supported treatments for PGD, after which screening could be made routine for widows.

## Introduction

In the aftermath of more than a decade of armed conflict and two large earthquakes affecting 8.5 million people, Nepal has a large population of widows (United Nations Country Team, 2007; Women for Human Rights, 2010). Widows in Nepal have high rates of depression, anxiety, post-traumatic stress disorder (PTSD), and suicidal ideation (Luitel *et al*., 2013; Basnet *et al*., 2018; Garrison-Desany *et al*., 2020). Suicide, the leading cause of death for Nepali women of reproductive age (Pradhan *et al*., 2011), is also strongly associated with prolonged grief disorder (PGD) in widows (Garrison-Desany *et al*., 2020).

PGD refers to the maladaptive grief response after individuals experience a major loss (Prigerson *et al*., 2009), such as the death of a husband. The 13-item Prolonged Grief Scale (PG-13) was developed to diagnose PGD according to the consensus criteria for a clinical diagnosis of PGD developed in 2009 (Prigerson *et al*., 2009). These consensus criteria became precursors of the PGD diagnostic criteria in the International Classification of Diseases 11 (ICD-11) (Killikelly and Maercker, 2017; World Health Organization, 2018) and the Diagnostic and Statistical Manual of Mental Disorders – 5th edition (DSM-5) (Prigerson *et al*., 2021). PG-13 uses the following criteria: individuals feel distress (i.e. longing or emotional pain) as a result of the experienced loss, symptoms have been experienced daily for at least 6 months following the loss, individuals experience at least five of the eight cognitive, emotional, and behavioral symptoms ‘quite a bit’ or ‘overwhelmingly’, and individuals experience impairment in functioning (Prigerson *et al*., 2009). Complicated Grief Treatment (Shear *et al*., 2005), as well as integrative cognitive behavioral therapy for prolonged grief (PG-CBT), individual CBT, and group-based CBT combined with exposure therapy were found to be effective treatments for PGD in high-income settings (Boelen *et al*., 2007; Bryant *et al*., 2014; Rosner *et al*., 2014).

Widows in Nepal are a stigmatized population who face substantial discrimination, low social status, economic insecurity, restricted living arrangements, and are often denied basic rights of mobility, nutrition, and healthcare (Coomaraswamy, 2005; Uprety and Adhikary, 2009; Surkan *et al*., 2015). These stressors and experiences may have profound effects on the mental and physical health of widows long after initial periods of bereavement (Haviland *et al*., 2014; Sabri *et al*., 2016; Kim *et al*., 2017; Hendrickson *et al*., 2018).

Despite this need for mental health services among widows, access to mental health services in Nepal is extremely low. Only 8.1% of people meeting the clinical cut-off threshold for depression had received treatment from any provider in the prior year in Chitwan district (Luitel *et al*., 2017). Not only is there a scarcity of mental health services in Nepal, but existing mental health resources are unequally distributed (Lund *et al*., 2012; Luitel *et al*., 2017), which creates barriers for rural and low-income populations in accessing care.

In order to respond to the unique needs of widows in Nepal experiencing PGD, increased screening is necessary. Accurate and reliable data on PGD among widows in Nepal can allow for the more efficient allocation of mental health resources and enable providers to have better understanding of the needs and vulnerabilities facing widows (Patel *et al*., 2008). In response to these detection, diagnosis, and treatment needs of widows, we conducted mixed-methods research to locally adapt and validate the PG-13 (Kim *et al*., 2020; Surkan *et al*., 2021). The newly adapted scale (PG-12/17-N) contains 12 original scale items and five new items reflecting symptomology in the local context (Table 1). PG-17-N demonstrated strong psychometric properties among widows in central Nepal (Cronbach's *α* = 0.93; sensitivity = 82%; and specificity = 74%) (Surkan *et al*., 2021).

**Table 1.** Prolonged Grief Scale adapted and validated for widows in Nepal (PG-12/17-N)

Item no.	English back translation	Nepali
1	In past 1 month, how many times did you feel that you needed your husband very much, you missed him very much, or you wished that he were with you?	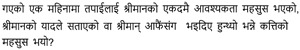
2	In past 1 month, how many times did you feel extreme pain, suffering (e.g. feeling of internal pains, feel like crying from deep within, extreme hurt, etc.) because of your husband's death?	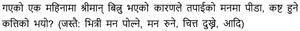
3	(Read question 1 or 2) Did you persistently have the aforementioned experiences/problems up to 1 year or daily even after 1 year of your husband's death?	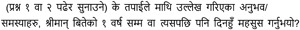
4	In past 1 month, how many times did you try to avoid things that reminded you of your husband? (e.g. not wanting to see your husbands’ clothes, photo, or anything associated with him; not wanting to go to the place where you had gone to with your husband; or not wanting to cook or eat the meal your husband liked; etc.)	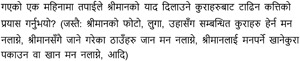
5	In past 1 month, how many times have you been shocked (e.g. you felt that you couldn't think at all or you have lost your senses) or confused because of your husband's death?	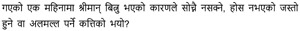
6	In past 1 month, how often have you been confused with your role (e.g. what to do next in your life) or who you are or felt that you don't know yourself?	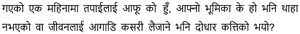
7	In past 1 month, how difficult has it been for you to accept or believe that your husband has passed away?	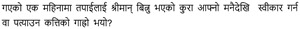
8	In past 1 month, how difficult has it been for you to believe in others because of your husband's death? (e.g. when doing financial transactions, traveling around, taking help from others)	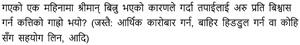
9	In past 1 month, how often have you felt very bitter (like unpleasant feeling, feeling down, feeling blank, or feeling frustrated) because of your husband's death?	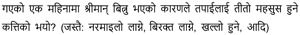
10	In past 1 month, how difficult has it been for you to forget the pain, grief of your husband's death and get back to your normal life? (e.g. running your household, educating your children, etc.)	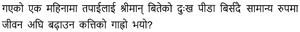
11	In past 1 month, how often have you felt that your life is incomplete or meaningless because of your husband's death? (e.g. feeling like there is nothing you can do by living or there is nothing to live for)	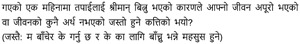
12	In past 1 month, have you had difficulty taking part in social functions or doing household chores? (e.g. not wanting to go to wedding or Bratabanda, not wanting to attend the meetings, assemblies organized locally)	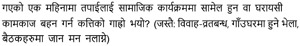
13	In past 1 month, how often have you been tense or worried about running your household, rearing and educating your children because of your husbands’ death?	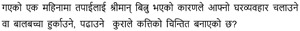
14	In past 1 month, how often have you had thoughts playing in the heart-mind or been thinking too much in past one month because of your husband's death?	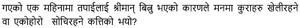
15	In past 1 month, how often have you been very worried, stressed, or restless because of your husband's death?	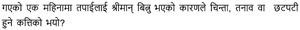
16	In past 1 month, how often have you felt that you are not interested in doing anything or capable of doing anything because of your husband's death?	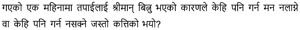
17	In past 1 month, how often have you felt insecure because of your husband's death? (e.g. scared to go the places where you used to go with your husband, scared if anyone would do anything or say something, etc.)	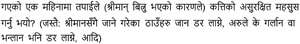

Items 1–12 from the original PG-13; items 13–17 are additional items specific to Nepali widows. See Kim *et al*. (2020) for details on scale adaptation and Surkan *et al*. (2021) for scale validation.

Although the PG-17-N is validated for use in Nepal to enhance the screening and treatment for PGD among widows, research is needed to understand how best this scale can be implemented. Given the scarcity of mental health human resources in Nepal, it is of particular importance that the feasibility of implementing the PG-17-N is examined in order to promote its uptake and usage among both specialists and lay health providers. This study aims to assess the feasibility of the implementation of PG-12/17-N and provide actionable recommendations for implementation by analyzing barriers and facilitators using the Consolidated Framework for Implementation Research (CFIR).

## Methods

### Study setting

Nepal is one of the poorest countries in South Asia (World Bank World Development Indicators, 2019). This study took place between June and August 2018 in Kathmandu valley and Chitwan district. Kathmandu is the capital of the country, where existing mental healthcare resources are concentrated. Chitwan, a district in Southern Nepal, was the implementation site of the PRogramme for Improving Mental Health CarE (PRIME).

PRIME developed a district mental healthcare plan (MHCP) for implementation and evaluation based on the Mental Health Gap Action Programme (mhGAP) by the World Health Organization (WHO), a guideline for the detection, diagnosis, and treatment of mental disorders in primary care settings (Lund *et al*., 2012; Jordans *et al*., 2016; Luitel *et al*., 2020). Prescribers were trained on pharmacological treatment of mental disorders, whereas non-prescribers were trained on psychosocial interventions (Upadhaya *et al*., 2020). Female community health volunteers (FCHVs) were trained to operate home-based care, community awareness programs, and mental health case detection (Upadhaya *et al*., 2020).

### Participants and recruitment

Study participants were recruited through the corresponding author's institute in Nepal, which is a non-governmental organization (NGO) that was the implementing organization of PRIME (Lund *et al*., 2012). Participants were purposefully sampled to include providers with varying levels of contact with widows in their programmatic work, their role in their organizations, educational background, and affiliation with the public healthcare system. The first and second authors contacted potential participants via email or phone and scheduled face-to-face interviews in a follow-up phone call. Twenty-five providers participated including: five staff members in NGOs that serve widows; five psychosocial counselors; seven government health workers (working in health posts, primary healthcare centers, or district hospitals); six psychiatrists or psychologists; and two FCHVs (Table 2).

**Table 2.** Characteristics of participants (*n* = 25)

Characteristics	*N*
Location	
Kathmandu	16
Chitwan	9
Years of experience^a^, mean (range)	10.7 (1–30)
Provider type	
NGO staff	5
Psychosocial counselor	5
Government health worker	7
Psychiatrist/psychologist	6
FCHV	2
Worked with widows	
Yes	19
No	6

NGO, non-governmental organization; FCHV, female community health volunteer.

a Two responses were missing.

### Interview process

Semi-structured interviews were conducted in a private space at the participants’ workplaces or the offices of the author's institute. The first author, a female identifying, US-based doctoral student visiting the corresponding author's NGO, conducted interviews with participants who preferred to use English, and the second author, a female identifying, Nepalese research assistant with a bachelor's degree in Public Health and prior experience in qualitative research, conducted interviews in Nepali with the first author present. At the beginning of the interview, participants were asked to review the PG-17-N. The interviewer explained that the five items at the end of the scale were being proposed as new items in order to capture symptoms specific to widows in Nepal. Once participants finished reviewing the instrument, they were asked a series of open-ended questions about selected constructs of the CFIR. The scale's scoring criteria were presented and explained to participants when scoring was discussed during the interview. The interviews were 30 min to an hour long and were digitally recorded and transcribed verbatim. The first and second authors individually made field notes and discussed entries while transcribing the interviews. Written informed consent was obtained prior to each interview.

### Data analysis

Constructs in the CFIR guided the design and analysis of this study. The CFIR is a summary framework of constructs that were identified as relevant to effective implementation of health services. Researchers can select a subset of constructs that are most relevant to their study from this framework (Damschroder *et al*., 2009). Thirteen constructs in four domains (instrument characteristics, individual characteristics, inner setting, and outer setting) were selected for this study.

The constructs in the domain of instrument characteristics relate to scale attributes (PG-17-N) that would influence its implementation, including the strength of evidence, relative advantage (over other scales), complexity, and cost. Individual characteristics refer to the characteristics of the individuals who implement the scale. This study focused on providers’ knowledge and beliefs about the scale and self-efficacy to use it, constructs inspired by individual-level behavior change theories, such as the theory of planned behavior (Ajzen, 1991). Constructs under the domain of inner setting refer to organizational characteristics that may facilitate or hinder the introduction of the scale (structural characteristics of the organization, culture, implementation climate, and readiness for implementation). The outer setting pertains to the structures that exist beyond each organization, such as perceived population needs, practices in other organizations, and external policies and incentives.

The transcripts were analyzed using both inductive coding and deductive coding based on the CFIR. First, the first and second authors did close readings of the transcripts. In the initial coding phase, the two authors applied codes based on the CFIR to the transcripts (13 selected constructs were used as codes) and generated more codes based on the data. After completing the initial coding of half of the transcripts, the two authors developed a codebook using recurring codes. The first author coded the remaining half of the transcripts according to the codebook, which was continuously updated throughout the coding process. The final set of codes was applied to all transcripts, and themes were extracted after reviewing all coded segments. The analysis was performed on Dedoose (version 8.0.35, Los Angeles, CA).

## Results

### Sample characteristics

Of the 25 providers, 16 were based in Kathmandu and nine in Chitwan. The providers in Kathmandu were policymakers, staff, and psychosocial counselors in NGOs, and psychiatrists and psychologists, whereas the providers in Chitwan included primary healthcare workers and FCHVs. Excluding two who did not provide a numeric estimate, providers in this study had an average of 10.7 years of experience (range 1–30 years). Most providers had direct contact with widows, except for six who were either policymakers or supervisors (Table 2).

### Instrument characteristics – perceived strengths

The most prominent strengths of the instrument discussed by providers were need, relevance, and easy and specific language. Most providers recognized a need for the scale in Nepal due to the lack of help and resources for widows. A policymaker pointed out that since the initiative to establish a mental healthcare system in Nepal is in its beginning stages, it would be difficult to focus resources on a specific group. However, the vulnerabilities of Nepali widows derived from social conventions and low social and economic status were widely recognized by providers.

Providers who previously had widowed patients recognized the symptoms in the instrument, especially in the newly added five items (items 13–17, Table 1), in their clients. They voiced that the items closely reflected the experiences of their clients. A psychosocial counselor in Chitwan was reminded of a client while reviewing the instrument.

‘I can relate these symptoms to her [the client] and her problems. These symptoms reflect her own words. […] The items mentioned in this scale are so relevant to her situation. These words exactly describe her feelings’. – Psychosocial counselor, Chitwan

The fact that the instrument was adapted for the Nepali cultural context and language differentiated the instrument from other scales providers used before.

‘We have been using scales prepared in Western society, so the language and other terms might not be relatable to our context. However, I think that all terms incorporated into this scale are used in Nepali society’. – Psychosocial counselor, Kathmandu

The original instrument (PG-12-N) was regarded by many providers as relevant to other bereaved populations in Nepal. The language in the scale was perceived to be easy enough for community members to understand. Since the instrument incorporates commonly used vocabulary and is based on symptoms as described directly by community members, counselors reported they would like to use the instrument in counseling sessions.

‘[Counsellors] do find this kind of scaling, questionnaire, to be more helpful when they want a more structured way…to know what's really going on with the client. It's really helpful’. – Psychosocial counselor, Kathmandu

Clinicians, on the contrary, who use instruments to diagnose and screen patients, valued the specific language in the scale. The specific language used to describe symptoms was perceived to be advantageous in differentiating PGD from depression.

‘We have been diagnosing such symptoms as depressive symptoms. But after reviewing the PGD scale, I can say that those depressive symptoms actually match with PGD. Though it contains fewer symptoms of depression, we still diagnosed them as depression’. – Public Health Inspector, Chitwan

### Instrument characteristics – perceived weaknesses

The instrument was perceived as being longer and having more complex response options and scoring criteria compared to other instruments. The scale most frequently used by providers was the Patient Health Questionnaire-9 (PHQ-9), and most questionnaires already in use have around 10 items. As the items in PG-12/17-N are detailed and include many examples, individual items are also longer than items in other scales. Primary care providers questioned whether they would have enough time to implement the PG-12/17-N.

'Since we have to look after many health problems in our health facility, it's very difficult to give lots of time to a single patient. So, if the items were short, it would be effective in our setting’. – Auxiliary Nurse Midwife, Chitwan

Some providers expressed that the frequency- and severity-based response options could be difficult for community members with low levels of education. Using two types of response options in a single scale created confusion for a few providers as well. Since the Prolonged Grief scale is a diagnostic tool, the scoring involves matching questions to their designated criterion and checking whether the responses meet the criterion. This scoring method was perceived to be more complex compared to the method used in many screening tools (i.e. sum each item score and determine clinical significance based on the total score). Regarding the content of the items, the three main perceived weaknesses were redundancy, overlap with depression symptoms, and lack of somatic symptoms. A few providers perceived there to be repetition of items within the scale. Other providers reported that the symptoms included in the scale, especially feeling down (item 9) and loss of interest (item 16), seemed consistent with depression. A couple of providers suggested it would be helpful to include somatic symptoms such as insomnia in the scale since they observe those symptoms frequently in their clients.

### Individual characteristics and inner setting – training and supervision need for implementation

Most providers showed confidence in using the scale but conditioned their confidence on having appropriate training and supervision available. Healthcare providers in Chitwan who received training to use other scales as a part of the district MHCP demonstrated higher self-efficacy and knowledge compared to providers who did not have mental health training.

Necessary training to use the scale identified by providers included basic mental health training and counseling techniques. Based on their prior training with PRIME, they suggested that basic understanding of mental health conditions allowed them to confront their stigma toward people with mental health issues.

‘We used to use bad words to denote them, such as “*psycho*”, “*pagal* (mad)”, and “*khuskeko* (lost mind)”. But after the training, we felt we were discriminating against them, and now we don't have such negative thoughts towards them. Nowadays, if I see such patients, I would ask if they are doing well or if they have any problem’. – Auxiliary Nurse Midwife, Chitwan

Providers also believed that understanding disordered grief in the context of other mental health conditions is important to prevent over-diagnosis of PGD.

'They [who use the scale] should have basic mental health training. And that is a must I think because they need to understand everything in context. If you just train them in Prolonged Grief Disorder, everything will be Prolonged Grief Disorder. So, they need to have a general idea (broad understanding) first’. – Psychiatrist, Kathmandu

Another suggestion was that the healthcare providers who use this scale should have training in counseling techniques such as building rapport, creating a safe environment, and providing psychological first aid. Although most providers did not perceive the items to be sensitive, they pointed out that as the topic of grief is emotional, respondents need adequate support when answering the questions.

The general opinion among providers was that psychosocial counselors with at least 6 months training are likely to have sufficient mental health knowledge and counseling skills to be able to implement the scale effectively. However, prescribers (not trained to provide counseling) and community-based health workers expressed confidence in implementing the scale, on the condition that they would receive a short training of 1 or 2 days. They believed that if they can use the scale and provide basic counseling, it would be beneficial since widows would not have to go through more referrals to get help.

‘If the patients go directly to the counsellors, then it would be okay, but since they come directly to us [prescribers], they expect treatment from us. If we got the training, it would be beneficial to the patients themselves’. – Public Health Inspector, Chitwan

‘We [community-based health workers] need training. Just like the counsellors at [NGOs], we also need such training that would help us do the counselling at our level. When we ask community people to visit counsellors, they don't go there. If only we were trained about counselling, we could treat women, old male/female, adults, adolescents, children, every segment of the population. The people in the community don't trust any unknown person, even if that person is a counsellor. They think that he/she might tell people in the community about their condition. If we could receive training, we could create a feasible and comfortable environment for them, and also refer them to the hospital’. – FCHV, Chitwan

A single training to use PG-12/17-N was, however, not deemed sufficient. Providers emphasized the value and necessity of constant supervision from psychologists and psychiatrists. There were two main reasons: first, PGD cases identified may not be handled or referred correctly without structured supervision, and second, providers themselves may need psychological support due to potentially strong emotions experienced while interacting with widowed women. Structured supervision was usually in place within private organizations, often in conjunction with doctors who practice in large hospitals. However, there was no built-in supervision structure in primary healthcare settings. Supervision had formerly been available in Chitwan but ceased as the PRIME activities were phased out. Many providers in Chitwan shared regret that they no longer had access to supervision.

### Outer setting – system-level requirements for implementation

In terms of external structures required for implementation of the scale, providers predominantly discussed detection and referral systems. The FCHVs were regarded as having a critical role in connecting widowed women in the community to the primary healthcare facilities. As FCHVs are trusted by other women in the community, primary care workers suggested training FCHVs to identify widowed women, use this scale, and provide counseling. Additionally, the importance of raising awareness of PGD and mental health in general was discussed. Providers based in Chitwan described having experienced that once there was sufficient community awareness about a mental health issue, patients visited primary healthcare facilities on their own for check-ups.

A system-level requirement to implement the PG-12/17-N that was perceived as lacking was a referral system for widowed women identified as at high risk for PGD. In most areas outside of Kathmandu, there is a lack of mental health and psychosocial support services, especially when higher-level care is required, with no specific mental health services for widowed women. Mental health services specifically for widows, however, were regarded as necessary given these women's unique set of vulnerabilities as well as perceived high needs. One psychosocial counselor mentioned the time constraints women face in accessing services.

'The widows are women, and they have other things, they have to work for their basic needs. That's why it might be difficult to approach daytime services’. – Psychosocial counselor, Kathmandu

## Discussion

This study qualitatively explored the feasibility of implementing the PG-12/17-N, a tool adapted and validated to measure PGD among widows in Nepal, by interviewing health service providers in Kathmandu and Chitwan. Providers recognized the need to implement the scale, citing widows’ socioeconomic vulnerability in Nepali society that increases their risk of mental disorders such as PGD. Providers perceived the main advantages of the scale to be cultural relevance, easy language, and inclusion of detailed and specific items. The complexity in response options and scoring, length, item redundancy, overlap with depressive symptoms, and lack of somatic symptoms were the perceived weaknesses of the scale. Providers also discussed that training, supervision, and a referral and detection system were required to implement the scale in Nepal. The lack of referral resources and evidence-based treatment of PGD are major barriers to widespread implementation of the scale.

Providers in this study perceived the PG-12/17-N to be an acceptable tool for widows in Nepal and reported the items were culturally appropriate, easy to understand, and specific to the situation of Nepali widows. Because mental health measurement scales are seldom developed specifically for populations in low- and middle-income countries (LMICs) (Ali *et al*., 2016), it is important that such scales are translated and adapted rigorously before implementation to avoid misclassification of disorders (Patel and Wesseley, 1998; Bass *et al*., 2007).

Counselors who have experience working with widows pointed out the lack of somatic symptoms in the scale. A quantitative study with Nepali widows reported that neurological complaints such as headaches, dizziness, numbness, and general weakness were associated more strongly with suicidal ideation than bodily pain (chest, back, or muscle pain) (Garrison-Desany *et al*., 2020). An earlier qualitative study also documented that Nepali widows who experienced somatic symptoms lasting for more than 1 year had other symptoms associated with disordered grief (Kim *et al*., 2017). The lack of somatic symptoms therefore is a limitation of the scale that should be addressed in future revisions and adaptations.

Study participants expressed that use of the PG-12/17-N should be accompanied by proper training. As task-shifting is identified as an effective approach to improve access to mental health services in LMICs (Fulton *et al*., 2011; Joshi *et al*., 2014), proper mental health training of community-based health workers and primary healthcare staff has been emphasized (Eaton *et al*., 2011). Studies have demonstrated that PGD is distinct from but correlated with major depressive disorder and PTSD (Prigerson *et al*., 2009; Boelen *et al*., 2010). One of the weaknesses of the PG-12/17-N reported by the participants was that some items seemed more consistent with depression than PGD, but this perception may be alleviated if information about depression and PGD is added to existing mental health training. Similarly, providers, especially those with lower levels of training and experience, commented that the items in the PG-12/17-N seemed similar and redundant. This finding suggests that training on the types of symptoms (emotional, cognitive, and behavioral) measured to assess PGD will be important to ensure providers can explain the items to respondents effectively. Considering the current low awareness about PGD among policy makers and providers, materials and initiatives to sensitize stakeholders about the need for PGD services are required to update existing mental health training with information about PGD and the use of the PG-12/17-N.

Along with mental health training, participants emphasized the necessity of counseling training for personnel who apply the PG-12/17-N. These recommendations are in line with the Guidelines for International Training in Mental Health and Psychosocial Interventions for Trauma Exposed Populations in Clinical and Community Settings in which competence in listening and other communication skills is listed as one of the first core curricular elements (Weine *et al*., 2002). Prescribers and FCHVs in our study expressed that they wish to receive counseling training to use the scale and be able to counsel. When prescribers and community-based healthcare workers such as FCHVs are trained to provide counseling, it will eliminate the need to make referrals to psychosocial counselors, which could result in patients not following up to get counseling. As reported by FCHVs in our study, patients, especially socially vulnerable patients such as widows, may have greater trust in primary and community-based healthcare workers.

Providers in this study expressed concerns about using the PG-12/17-N either without the capacity to provide care or refer patients to appropriate care. One of the major challenges is the lack of evidence for treatment of PGD in Nepal and other LMICs. Wilson and Jungner's principle of screening (Wilson *et al*., 1968) states that screening should be conducted when facilities for diagnosis and treatment are available. Screening without treatment is unethical and most national health systems recommend against this (MacMillan *et al*., 2005; National Collaborating Centre for Mental Health, 2010; Thombs *et al*., 2012; Siu *et al*., 2016). Perceived lack of treatment and available resources can also deter use of and engagement in healthcare services (Bhardwaj *et al*., 2020). Thus, prior to the implementation of the PG-12/17-N, providers should be able to identify appropriate referral avenues for widows (Hanlon *et al*., 2010; Eaton *et al*., 2011). Currently, beside at the limited number of tertiary hospitals with psychiatric services, there is no evidence-based treatment for PGD in Nepal.

Trials are first needed to determine what treatments will be effective in LMICs. Complicating factors are that PGD is more often than not co-morbid with other psychiatric disorders, with PGD associated with on average 2.5 other psychiatric disorders (Rosner *et al*., 2014). Therefore, treatment would need to focus on multi-morbidity. In addition, standard cognitive behavioral techniques have been shown to be inferior to approaches which use exposure therapy (Bryant *et al*., 2014). Exposure therapy is a more complex psychological treatment that has been integrated into only a few of the therapies that are widely used in global mental health, none of which have been evaluated in Nepal. Therefore, rather than screening for PGD, it would be preferable to screen for commonly comorbid conditions that do have evidence-based treatments in Nepal, notably depression (Jordans *et al*., 2019). Therefore, as a first step, the Prolonged Grief scale could be used as an outcome measure in clinical trials of treatments for PGD. Then, once evidence-based treatment is regularly available, the tool (PG-12/17-N) could be regularly used.

Additional concerns for its future use include length. Primary healthcare providers in this study expressed that the length of the PG-12/17-N would be a barrier to its use in routine primary healthcare visits. When primary healthcare staff is already busy, they find it difficult to engage in tasks that require additional time (Eaton *et al*., 2011). On the contrary, psychosocial counselors in the non-governmental sectors identified the detailed and specific questions in PG-12/17-N as the strength of the scale since the questions help them structure their counseling sessions and ensure they do not miss any relevant symptoms. This finding suggests that PG-12/17-N in its current form can be particularly helpful to those who provide counseling to widows, rather than for providers in the primary healthcare setting.

To facilitate future use of the scale for research aimed to develop evidence-based treatments and ultimately for use in the primary healthcare system, a brief version of the PG-12/17-N scored based on the sum of item responses can be developed as a screening tool to be administered prior to the use of the full PG-12/17-N. A previous study in Nepal suggested screening patients with two local idioms of distress first and proceeding to the items in PHQ-9 only when patients answer positively to the two screening questions. The study found that this approach reduced false positives by 18% and the number of patients requiring PHQ-9 screening by 50% (Kohrt *et al*., 2016). At least one of the items used to screen patients could include somatic symptoms, which providers reported were common in bereaved women but were not included in the PG-12/17-N. Future research that uses item response theory can provide insight to shorten the scale and improve its usability.

This study utilized the CFIR, which systematically guided the understanding of facilitators and barriers to implementation of the PG-12/17-N (Keith *et al*., 2017). Moreover, we involved mental health providers at various skill levels in both public and private sectors. By including providers who directly interact with widows, who make policy decisions, and both with and without mhGAP training, we were able to capture a wide range of perspectives that influence what kind of mental health services widows experience. Another strength of the study is that it included providers who were familiar with the recent policy changes from the implementation of the PRIME district MHCP in Chitwan. This study was thus able to highlight the facilitators and barriers to implementation of the PG-12/17-N in the context of the most current mental health policy environment in Nepal.

A limitation of this study was that providers were only recruited from Kathmandu and Chitwan. Although inclusion of Chitwan-based providers provided a unique perspective based on what the primary care-based mental health services will develop into in other parts of the country as the MHCP-based government mental health policy continues to roll out throughout Nepal, the current implementation contexts in more under-resources parts of Nepal were not the primary focus of this study. Some study participants had experience working in more remote parts of the country and spoke from their cumulative experiences. Chitwan was also similarly under-resourced prior to PRIME, and most Chitwan-based providers were able to compare their previous context and current context (after PRIME) when discussing the implementation of the PG-12/17-N.

## Supplementary material

For supplementary material accompanying this paper visit http://dx.doi.org/10.1017/gmh.2021.29. click here to view supplementary material
